# Correlation of Kidney Lenght and Body Parameters in CT Scans

**DOI:** 10.1590/S1677-5538.IBJU.2026.9901

**Published:** 2026-01-29

**Authors:** Ana Raquel M. Morais, Carla M. Gallo, Luciano A. Favorito, Francisco J.B. Sampaio

**Affiliations:** 1 Grupo Fleury Rio de Janeiro RJ Brasil Grupo Fleury, Rio de Janeiro, RJ, Brasil; 2 Universidade do Estado do Rio de Janeiro Unidade de Pesquisa Urogenital Rio de Janeiro RJ Brasil Unidade de Pesquisa Urogenital - Universidade do Estado do Rio de Janeiro - Uerj, Rio de Janeiro, RJ, Brasil

**Keywords:** Anatomy, Tomography, X-Ray Computed, Body Mass Index

## Abstract

**Purpose:**

To analyze the renal length in patients submitted to computed tomography (CT scans) and compare it according to age, gender, laterality and body parameters like height, weight and Body Mass Index (BMI).

**Methods and Methods:**

We analyzed 74 patients (148 kidneys) submitted to CT scans and evaluated renal length in centimeters, gender, height, weight and BMI. The abdominal CT scans acquisition and image analysis was done using 16 and 64 slice multidetector computed tomography (MDCT) scanners to perform multiplanar reconstructions (MPR) and measure the kidney length (KL) in coronal plane. The statistical analysis was performed with the GraphPad Prism software (Version 9.2.0).

**Results:**

The 74 patients analyzed (28 Males/37.83% and 46 females/62.17%) presented mean age of 54.1 years-old, right kidney length between 8.4 to 13.1cm (mean=10.79) and left kidney length between 8.3 to 13.1cm (mean=10.97). The kidney length on both sides was significantly greater in male sex (p<0.001). The length of the left kidney was significantly greater than that of the right kidney (p=0.017). The linear regression analysis showed non-significant correlation between both right kidney length and positive correlations between kidney length and BMI, weight and height.

**Conclusions:**

CT scan accurately assessed renal length. We observed that renal length was greater in males and in the left side. Weight, age, height and body mass index showed a positive correlation with kidney length.

## INTRODUCTION

Knowledge of the normal sizes of the kidneys is important to radiologists and urologists when assessing the diagnosis and follow-up of renal diseases, using ultrasound scan (US) (1, 2). Renal length (kidney length) is an important parameter used in the clinical evaluation of renal growth and abnormalities and to estimate the renal cortex volume in chronic kidney disease (3–5). The kidney size can be affected by several diseases (6).

Renal size estimation most commonly incorporates renal length, renal volume and cortical thickness (3, 6, 7). The kidney length estimation is more solid because of its simple reproducibility, but the renal volume estimation is more precise (3, 6, 7).

During partial nephrectomies to treat kidney tumors the study of renal measurements and volume is very important and has great impact to surgical planning (8). The renal length is influenced by the overall body parameters, including age, height, weight and body mass index (BMI) (8–10). A few studies have been carried out on the normal dimensions of kidney size around the world (1, 2, 5, 11).

To our knowledge there are no studies that analyzed the length of the kidney with the measurements taken during CT scans and comparing the measurements with body parameters. Our hypothesis is that kidney length does not have important variations with age, genders, height, weight and BMI. The objective of the study was to analyze the kidney length in patients submitted to CT scans and compare it according to gender, age, laterality and body parameters.

## MATERIAL AND METHODS

This study was approved by the Ethical Committee on Human Research, of our institution with the number (IRB: 76133 223.1.0000.5259) and we confirm that all methods used in this paper were carried out in accordance with relevant guidelines and regulation. The study has also been registered in the Brazil Plataform, Ministry of Health, National Health Council, National Research Ethics Commission for studies with human beings. We confirm that all methods used in this paper were carried out in accordance with relevant guidelines and regulation in compliance to the declaration of Helsinki.

We analyzed 74 patients (148 kidneys) submitted to abdominal computed tomography (CT) for various clinical indications in our institution between April 2024 and May 2025. We included patients up to 150 kg and evaluated the renal length in centimeters, age, gender, height, weight and Body Mass Index (BMI). We excluded patients with kidney anomalies, previous kidney surgeries, tumors and cysts; patients with urinary obstructive factors and patients with kidney Infection and renal failure.

**Figure 1:**
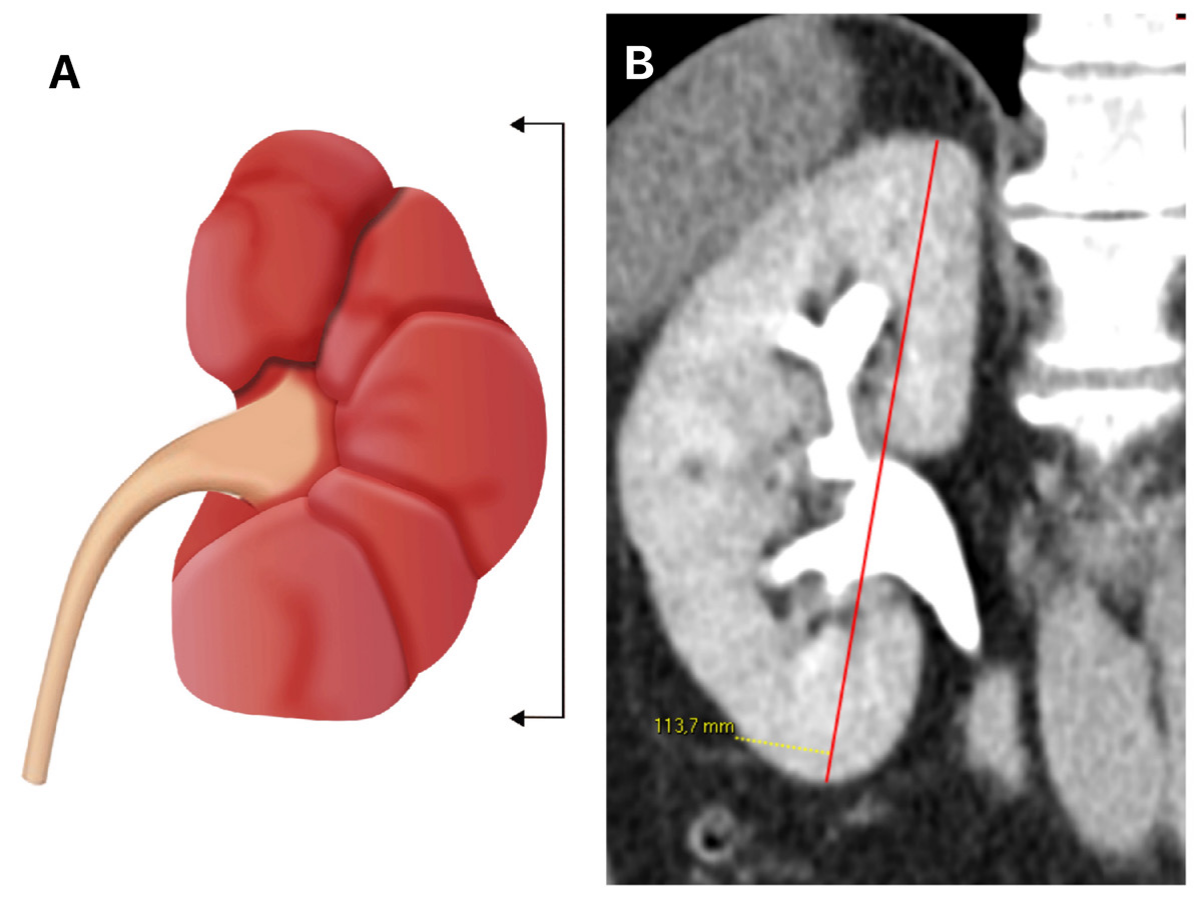
Kidney Length measurement.

The abdominal CT scans and image analysis was done using 16 and 64 slice multidetector computed tomography (MDCT) scanners. The reconstruction slice thickness was 1.25mm and the contrast used was the Henetix® 350 /Contrast dose 1.3 mL/ Kg. An experienced radiologist evaluated the images acquired in the excretory phase using the commercially available Picture Archiving and Communication System software (PACS) Carestream® to perform multiplanar reconstructions (MPR) and measured the kidney length (KL) in coronal plane. The KL measurement was taken three times by the single observer (12, 13), and the average was calculated (Figure-1).

A) Schematic drawing of kidney length measurement and B) Kidney length measurement in coronal plane (red line) in a CT scan image acquired during the excretory phase.

### Statistical Analysis

All parameters were statistically processed and graphically described. The Shapiro-Wilk test was used to verify the normality of the data. Student-t test was used for comparison of quantitative data (p < 0.05), and the level of significance was adjusted by the correction of Bonferroni. Simple linear correlations were calculated for renal length according to patients age, height, weight and BMI.

Simple linear correlations (r² values less than 0.4 reflect very weak correlation, while r² between 0.4 and 0.7 reflect moderate correlation and r² greater than 0.7 indicates strong correlation) were calculated for kidney measurements, according to biometric parameters. The statistical analysis was performed with the GraphPad Prism software (Version 9.2.0).

## RESULTS

The 74 patients analyzed (28 Males - 37.83% and 46 females - 62.17%) presented ages between 19 and 79 years-old (mean=54.1), weight between 50 and 121kg (mean=74.35), height between 150 to 186cm (mean=166.6), body mass index between 15.2 to 38.19 (mean=26.72), right kidney length between 8.4 to 13.1cm (mean=10.79) and left kidney length between 8.3 to 13.1cm (mean=10.97). The summary of findings of the patients analyzed is reported in Table-1. The renal length on both sides was significantly greater in male sex (p<0.001). The length of the left kidney was significantly greater than that of the right kidney (p=0.017).

**Table 1:** The table shows the parameters of our sample (74 patients) analyzed in the present study.

Parameters	SEX	Sample	Mean	SD	IQR
**Age(years-old)**	F	46	56.7	15.168	24.50
	M	28	49.9	14.496	22.50
**Weight (kg)**	F	46	68.5	11.833	13.50
	M	28	83.9	13.337	13.50
**Height_(cm)**	F	46	161.6	6.234	9.75
	M	28	174.8	7.493	7.25
**BMI**	F	46	26.2	3.857	5.02
	M	28	27.6	4.725	6.13
**RKL (cm)**	F	46	10.5	0.861	1.07
	M	28	11.3	0.905	1.33
**LKL (cm**	F	46	10.6	0.939	1.17
	M	28	11.6	0.710	1.20

BMI = body mass index; RKL = right kidney length; LKL = left kidney length; SD = Standard deviation; IQR = Interquartile range

**Figure 2:**
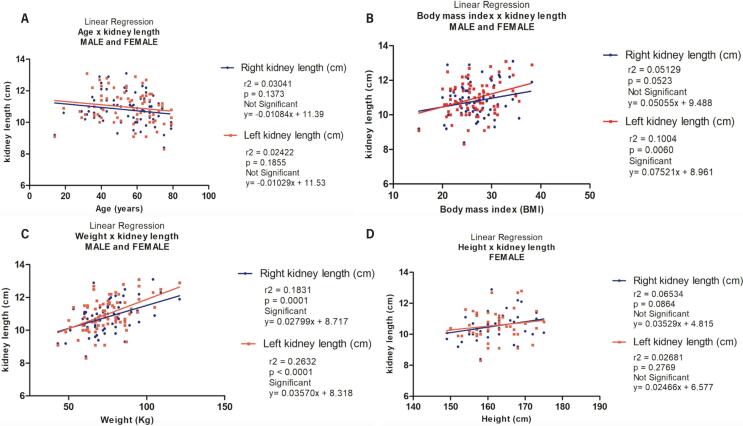
The figure shows the linear regression analysis comparing the biometric data with renal length in CT scans.

The linear correlation comparing renal length and the biometric parameters analyzed were assessed (Figure-2). The linear correlation comparing renal length and age were positive, but the r² values less than 0.4 in the right and left side indicates a very weak correlation. The linear regression analysis shows non-significant correlation between both right kidney length (r2=0.03041, p=0.1373, y=-0.01084x + 11.39) and left kidney length (r2=0.02422, p=0.1855, y=-0.01029x + 11.53) with age.

A) Renal Length X Patients Age: The linear regression analysis shows non-significant correlation between both right kidney length (r2=0.03041, p=0.1373, y=-0.01084x + 11.39) and left kidney length (r2=0.02422, p=0.1855, y=-0.01029x + 11.53) with age; B) Renal Length X Body Mass Index (BMI): the linear regression analysis shows non-significant correlation between right kidney length and BMI (r2=0.05129, p=0.05123, y=0.05055x + 9.488) and a significant correlation between the left kidney length and BMI (r2=0.1004, p=0.0060, y=0.07521x + 8.961); C) Renal Length X Patients Weight: The linear regression analysis shows a significant correlation between right kidney length and weight (r2=0.1831, p=0.0001, y=0.02799x + 8.717) and a significant correlation between the left kidney length and weight (r2=0.2632, p<0.001, y=0.03570x + 8.318). D) Renal Length X Patients Height: the linear regression analysis shows a significant correlation between right kidney length and height (r2=0.1703, p=0.0003, y=0.04192x + 3.814) and a significant correlation between the left kidney length and height (r2=0.1926, p<0.001, y=0.04743x + 3.070).

The linear correlation comparing renal length and BMI were positive, but the r² values less than 0.4 in right and left side indicates a very weak correlation. The linear regression analysis shows non-significant correlation between right kidney length and BMI (r2=0.05129, p=0.05123, y=0.05055x + 9.488) and a significant correlation between the left kidney length and BMI (r2=0.1004, p=0.0060, y=0.07521x + 8.961).

The linear correlation comparing renal length and height also indicates a weak correlation. The linear regression analysis shows a significant correlation between right kidney length and height (r2=0.1703, p=0.0003, y=0.04192x + 3.814) and a significant correlation between the left kidney length and height (r2=0.1926, p<0.001, y=0.04743x + 3.070).

The linear correlation comparing renal length and weight also indicates positive correlation. The linear regression analysis shows a significant correlation between right kidney length and weight (r2=0.1831, p=0.0001, y=0.02799x + 8.717) and a significant correlation between the left kidney length and weight (r2=0.2632, p<0.001, y=0.03570x + 8.318).

## DISCUSSION

The kidneys are located at the retroperitoneum, in contact with the major psoas muscle in each side, and therefore their longitudinal axis parallels the oblique direction of the psoas. The adult kidney has a length around 12 centimeters (cm) that vary among ethnic groups and are influenced by other factors such as body size, age and sex (14). The most accurate measurement of kidney size is estimated by the kidney volume, which could be correlated with biometric parameters like height, weight, and total body area (15, 16). The precise calculation of renal volume with US would be inappropriate, since it can underestimate the renal volume (17). Kidney volume is a better approximation of kidney size than renal length because of the shape of the kidney, but it is technically more demanding and needs four measurements in two different planes (15, 17, 18).

Renal length may not be an absolute predictor of overall kidney size, perhaps due in part to the fact that it measures only a single renal dimension, which may be associated with several inconsistencies and individual variations (17, 18). The renal volume has been emphasized by several authors as a true predictor of kidney size, however the renal length is easier to measure and is considered a good parameter for evaluating renal pathologies (19, 20).

Estimation of renal size could be a crucial step in the evaluation and treatment of several diseases, including cystic kidney diseases, chronic renal failure and renal masses (21, 22). US is the standard imaging modality in the investigation of renal diseases due to its noninvasive nature and easy availability; however it depends on the operator. CT scans are more commonly used for staging and characterization of renal cancer and could be an option for renal volume estimation because it has better accuracy in measurements (23).

Different studies have shown a correlation between renal measurements obtained via ultrasound and somatic parameters. In this paper for the 1st time in literature we studied the correlations between the anthropometric estimations with renal length using CT scans. Capaccioli and collegues (24) found a good correlation between age and kidney length. In a study with a larger sample size, Safak and collegues (25) examined 712 healthy school-age children and reported that weight best correlated with kidney length. The US reports are inconsistent on the relationship between body indices and renal morphology; some studies have found negative correlations between age and renal size (15,17,26). In our paper the linear correlation comparing renal length and age were positive, with a very weak correlation.

Several studies show that the renal length measurements were significantly associated with body mass index (BMI), weight, and body surface area (5, 8, 9). Emamian and collegues (27) reported evidence linking kidney length to weight, height, and body surface area, and BMI, while Han and Babcock (28) reported a correlation between kidney length and BMI. In our study, correlations show that weight, height, and BMI are significantly associated with renal length, especially for the left kidney. BMI and height were significant predictors for the right kidney, while sex and BMI were significant for the left kidney. The results indicate significant differences in kidney length between sexes, with men having larger kidneys, which may be related to anthropometric differences, such as greater weight and height.

Some studies have demonstrated no significant differences between the left and right kidney sizes. However, in our study the left kidney was significantly larger than the right kidney, which is similar to what was reported in other studies (5, 8, 9, 17, 26). The significant difference between the length of the right and left kidney suggests anatomical asymmetries, consistent with previous studies. The reasons to explain the difference between the length of the right and left kidney would be that the spleen smaller size compared to the liver, so the left kidney has more space to grow and the fact that the left renal artery is shorter than the right one, so increased blood flow in the left renal artery may result in a relatively increased size of the left kidney (14).

We should mention some limitations of this study: small sample size, study carried in a single institution, and we did not measure the renal volume.

In conclusion the CT scan accurately assessed renal length. We observed that kidney length was greater in males and in the left side. Weight, age, height and body mass index showed a positive correlation with kidney length.

## Data Availability

All data generated or analysed during this study are included in this published article

## ABBREVIATIONS

CT: = computed tomography
BMI: = body mass index
MDCT: = multidetector computed tomography
PACS: = Picture Archiving and Communication System software
MPR: = multiplanar reconstructions
KL: = kidney length
US: = Ultrasound Scan
